# Effects of Salt and pH on Aerobic Plate Count Methods for Low‐Water Activity Foods

**DOI:** 10.1111/1750-3841.71502

**Published:** 2026-11-11

**Authors:** Waraporn Mahlman, Jayne Stratton, Reka Howard, Tyler Wiederich, Tai Ha, Andreia Bianchini

**Affiliations:** ^1^ Department of Food Science and Technology University of Nebraska‐Lincoln Lincoln Nebraska USA; ^2^ Department of Statistics University of Nebraska‐Lincoln Lincoln Nebraska USA; ^3^ Nebraska Department of Agriculture Laboratory Lincoln Nebraska USA

## Abstract

The aerobic plate count (APC) method measures aerobic microorganisms in food that can grow on plates under specific conditions. This study assessed the impact of salt and pH on three APC methods –3M Petrifilm AC, pour plate and spread plate—for enumerating bacteria in a low‐water activity model food (pancreatin powder). In high microbial load samples, no significant differences were observed among the three methods. However, salt concentration and pH significantly affected all methods. In low microbial load samples, the methods showed significant differences, with a significant interaction between salt and pH. The pour plate and spread plate methods showed better bacterial recovery than 3M Petrifilm AC, especially with decreasing pH and increasing salt concentration. These findings are important for selecting the best method to assess the microbiological quality of low‐water activity foods with varying salt and pH levels. This study was conducted using a low‐water activity model food; therefore, further research is needed to validate these findings across a wider range of commercial low‐water activity food matrices.

## 1 Introduction

The 3M Petrifilm AC plate is widely used by many laboratories, not only in food manufacturing but also by government agencies. Validation has been conducted across various categories and types of food and environmental samples by either 3 M scientists or subcontractor institutes, academia, and regulatory agencies. A comprehensive list of validated food matrices for 3M Petrifilm AC is provided in Tables S1 and S2.

The review of the current literature as evidenced by the data shown in Tables S1 and S2 reveals that there is still a lack of method validation for using 3M Petrifilm AC to enumerate aerobic bacteria in specific food matrices. Even though some food products with low water activity, such as wheat flour, pasta, milk powder, and spice powders like pepper, garlic, and basil, have undergone method validation (3M Petrifilm company technical document 2019), methods for low water activity food matrices containing varying salt contents and pH levels have yet to be validated.

Numerous types of food contain elevated levels of salt; for instance, spice mixes may have salt concentrations as high as 30% and above (FitAudit, 2023). Most food products consist of mixtures of several ingredients, with salt being a staple ingredient used for both flavor enhancement and preservation purposes (Henney et al. 2010). The presence of salt can significantly impact the growth of microorganisms, particularly during enumeration tests such as the aerobic plate count (APC). Elevated salt concentrations or pH variations can inhibit the growth of certain types of organisms, potentially leading to inaccuracies if inappropriate methods are used.

A previous study indicated that salt concentrations exceeding 11.2% adversely affected the 3M Petrifilm AC method (Mahlman 2024), leading to significantly lower counts when compared to the standard pour and spread plate methods. This discrepancy may result from not all microorganisms present in the sample being able to thrive on the 3M Petrifilm AC plates under high salinity conditions. Environmental adaptation likely plays a pivotal role in microbial growth. According to Li et al., elevated salt concentrations (above 10%) inhibit the growth of *E. coli*, diminishing glucose consumption and glycogen accumulation within the bacteria (Li et al. 2021). When susceptible microorganisms encounter saline conditions, the cell wall loses its protective capacity against elevated osmotic pressure, leading to the outflow of water from the cell. Consequently, the protoplasm retreats from the cell wall, culminating in cell death (LibreTexts Biology, 2023). The elevated salinity diminishes the total microbial population since only halophilic or halotolerant microorganisms can endure such conditions (Ventosa et al. 2015). Microorganisms within this category include *E. coli*, *S. aureus*, *P. aeruginosa*, and various *Vibrio* species (Kushner 1978).

Just as salt plays a crucial role in the recovery and enumeration of microorganisms, pH also significantly influences bacterial growth. Most microorganisms exhibit an optimal pH growth range of approximately 6.5 to 7.5. Excessively acidic or alkaline conditions can lead to growth inhibition (Supriya, 2023).

Due to the influence of pH and salt on microbial growth, microorganisms tend to thrive in environments that suit their specific requirements. Consequently, when plating a food sample for bacterial enumeration on an agar plate, the pH and salt conditions will impact the total count of microorganisms that grow on the plate. These factors play a crucial role in determining the suitability of methods for microbial recovery and enumeration. Therefore, the goal of this research was to evaluate the influence of salt and pH on the enumeration of aerobic bacteria by 3M Petrifilm AC, pour, and spread plate methods, and to assess the robustness of these methods using a low‐water activity model food.

In this study, pancreatin powder was used as a model food matrix because commercial formulations may contain varying levels of salt, and the matrix allows for the controlled adjustment of pH and salt concentrations with minimal background interference. Although pancreatin powder is primarily marketed as a dietary supplement, its consumers may represent a vulnerable population, particularly individuals who have undergone partial or complete pancreatic resection and rely on pancreatic enzyme supplementation. Consequently, ensuring the microbiological quality and safety of pancreatin products is of particular importance. Therefore, selecting an appropriate aerobic plate count (APC) method is critical for obtaining accurate microbiological results under these stress conditions.

## 2 Materials and Methods

### 2.1 Sample Preparation

Pancreatin powder, which is a food supplement with low water activity and varying levels of salt content, was chosen to be the model food for this research. Generally, the pH of pancreatin powder is close to neutrality; therefore, it allows for the adjustment of the pH to varying levels as needed. The pancreatin powder used in this study was obtained from a food ingredient manufacturer based in the U.S. The samples included several commercial batches with 0.05% sodium (5 mg/g) and an average pH of 6.0. The different batches were pre‐assessed using traditional enumeration spread plate methods (Salfinger and Tortorello 2015), and based on the aerobic plate count results, were divided into two groups: high microbial load (10^4^ CFU/g and higher) and low microbial load (10^3^ CFU/g and lower). Samples falling into the high or low microbial load criteria were then combined to create three subgroups of homogeneous samples of each type. These homogeneous samples, representing high and low contamination, were then prepared according to the experimental design for this research. Therefore, sub‐samples had their pH and salt levels adjusted by adding sodium chloride and citric acid (both in powder form) until the target values required by the experimental design were reached.

Sodium chloride (CAS 7647‐145‐5) used for adjusting the salt content was obtained from VWR (VWR—Pennsylvania, USA), while citric acid (99+%, CAS 77‐92‐9) used for pH adjustment was acquired from Thermo Scientific (Thermo Scientific—Massachusetts, USA). The amount of sodium chloride and citric acid added to each sample to achieve the target values of pH and salt concentration are presented in Table 1.

**TABLE 1:** Amount of sodium chloride and citric acid added to each sample to achieve the target values of pH and salt concentration in pancreatin powder.

Target values		Amount added	
NaCl (mg/g)	pH	NaCl (g)	Citric Acid (g)
25.0	4.5	0.20	0.60
68.0	3.4	0.54	2.00
68.0	5.6	0.54	0.10
162.5	3.0	1.57	3.00
162.5	4.50	1.57	0.50
162.5	6.0	1.57	0.00
257.0	3.4	2.51	1.50
257.0	5.6	2.51	0.05
300.0	4.5	2.95	0.35

### 2.2 Determination of pH

Because the pancreatin samples were in a powder form, sterile water was added in a ratio of 1:2 w/w to enable pH measurement. Subsequently, the samples were mixed for 2 min using the Smasher (bioMérieux—France) blender. The pH of the sample was then measured using a pH meter (Oakton—South Carolina, USA).

### 2.3 Determination of Salt (NaCl) and Sodium Content

Salt and sodium quantification in pancreatin samples were carried out in triplicate using an ICP‐OES method with the help of a Thermo iCAP 6300 ICP‐OES Dual View (Thermo Fisher Scientific—Massachusetts, USA). The operating conditions for the equipment included: Plasma Setting at RF (Radio Frequency) Power 1150 W, auxiliary gas flow at 0.5 L/min, and nebulizer gas flow at 0.70 L/min. The iTEVA software (iTEVA Software Manual 2009) was utilized for data analysis, and wavelengths of 300.237 and 589.592 nm were employed for sodium quantification (Thermo iCAP 6300 ICP‐OES Dual View and the ICP‐OES operating manual, 2022).

All chemicals used for salt and sodium quantification were reagent grade. Microwave vessels were washed and soaked in 10% nitric acid, rinsed with distilled water, and dried before use. The samples were digested using a microwave digester model MARS 6 (CEM Corporation—North Carolina, USA), in concentrated HNO_3_: HCl (9:3 v/v) solution. Samples of approximately 1.0 g were pre‐digested with 9 mL of concentrated HNO_3_ for 2 h. Then 3 mL of concentrated HCl was added, vessels were closed, mixed, put in the microwave carousel, and digested per the following microwave digestion method: Power 1030–1800 W, ramp time of 20–25 min to 200°C, temperature hold time of 15 min at 200°C, and cooling time of 20 min. The resulting solutions were cooled, transferred to 100 mL plastic volumetric flasks, and brought to volume with distilled water. The sodium content was then determined by ICP‐OES.

Working standard solutions were prepared by dilution of a sodium stock standard solution to the desired concentration with HNO_3_: HCl (9:3 v/v) in volumetric plastic flasks to complete a 5‐point standard curve. The range of the calibration curve was selected to be inclusive of the expected concentration of sodium in the samples under evaluation. The correlation coefficient (*r*) of standard solutions and signal‐to‐noise obtained for the sodium standard curve was 0.999 (at 330.237 nm) and 0.999 (at 589.592 nm). Results were reported as sodium and NaCl concentrations in mg/g of sample.

### 2.4 Enumeration Of Aerobic Bacteria

To enumerate aerobic bacteria in the pancreatin powders, 10 g from each sample, prepared to achieve the target levels of salt and pH, were weighed into a sterile stomacher filter bag (Whirl‐Pak—Wisconsin, USA). The powder mixture was combined with 90 g of Butterfield's phosphate‐buffered dilution water. The sample was then stomached at a normal setting for 2 min using the Smasher (bioMérieux—France) blender. Following the stomaching step, serial dilutions were carried out to achieve 10^−1^, 10^−2^, 10^−3^, 10^−4^, 10^−5^, and 10^−6^. Each sample was divided into three sub‐units, and each sub‐unit was analyzed by all three enumeration methods. Each sample served as independent experimental replicates.

The pour plate method was carried out according to the method specified in FDA‐BAM Chapter 3. The first step included preparing Standard Method Agar (SMA) media (3 M media‐ St. Paul, USA) and tempering it at 45–50°C. Subsequently, 1 mL of each sample dilution was transferred into a Petri dish, mixed with 12–15 mL of molten media, and gently swirled to ensure thorough mixing. The plates were then allowed to cool before being placed into an incubator.

The spread plate method was performed according to the Compendium of Methods for the Microbiological Examination of Foods (Splittstoesser and Vanderzant 1992). To increase the sensitivity of the method, 1 mL from the 10^−1^ dilution sample was transferred onto three agar plates by dispensing 333 µL onto each plate followed by spreading with a sterile L‐spreader. The agar plates used were prepared ahead of time with 12–15 mL of agar in each plate. The results from all three plates were counted, and the sum was reported as the counts associated with the 10^−1^ dilution. From other serial dilutions, 100 µL was dispensed and evenly spread onto each plate.

For aerobic bacteria enumeration using the 3M Petrifilm Aerobic Count (AC) plates, manufacturer instructions were followed. In brief, once dilutions were prepared as previously described. One mL of each sample dilution was dispensed onto the plate, followed by sample spreading with the help of a 3M Petrifilm spreader to distribute the sample evenly.

All plates were then incubated at 35 ± 2°C for 48 ± 3 h. Following the incubation period, colony‐forming units growing on each plate were enumerated for each method under evaluation. Enumeration procedures outlined in the Compendium of Methods for the Microbiological Examination of Foods (Salfinger and Tortorello 2015) were followed. For those samples presenting very low contamination levels, an estimated count was obtained from plates that had less than 25 colony‐forming units.

### 2.5 Statistical Analysis

Experiments were conducted to assess the influence of pH and salt concentration on bacterial aerobic counts in two groups of distinctive samples: with high and low microbial load. Both experiments were conducted in the same manner. For each experiment, nine sub‐samples of the pancreatin powder (with high or low microbial load) were adjusted to achieve specific combinations of salt concentration and pH levels. These combinations were determined through a central composite design and are described in Table 2. Each sub‐sample was further divided into three sub‐units to then be plated using the 3M Petrifilm AC method, as well as pour and spread plate methods.

**TABLE 2:** Independent variables and their levels used for the central composite rotatable design.

Independent variables	Levels of coded variables				
	−*α*	Low	Medium	High	+*α*
	−1.41	−1	0	+1	+1.41
NaCl (mg/g)	25.0	68.0	162.5	257.0	300.0
pH	3.0	3.4	4.5	5.6	6.0

The level of each variable was established based on a preliminary evaluation of the salt and pH composition of commercially available spice blends. These ranges were selected to ensure that the study encompassed the broad spectrum of salt concentrations encountered in foods rather than being limited to pancreatin powder alone. The distance of the axial point from the center point was ±1.41 and calculated from equation *α* = (2*^n^*)^1/4^ where n is the number of variables.

The pH and NaCl ranges used in the central composite design (CCD) were established based on a preliminary survey of commercially available spice blends, which exhibited wide variability in acidity and salt content. Although pancreatin powder was used as the model matrix, it served as a controlled carrier system in which pH and NaCl levels could be systematically adjusted. Therefore, the CCD levels were selected to represent the range of conditions encountered in spice products and other low‐moisture foods rather than the native composition of pancreatin powder itself.

The statistical design used for this study was a split‐plot design where the main plot factor was a quantitative x quantitative CCD considering the salt and pH levels. The split‐plot factor was the method, which was treated as a qualitative factor. The statistical model used for the data analysis was:

$$\begin{array}{ccc} \;Y_{ijk} & = & \beta_{0}+\beta_{1}S_{i}+\beta_{2}P_{j}+\beta_{12}\left(S_{i}\;\times P_{j}\right)+W_{ij}+\beta_{3}M_{k}\\ & & +\;\beta_{13}\left(S_{i}\times M_{k}\right)+\beta_{23}\left(P_{j}\times M_{k}\right)+\beta_{123}\left(S_{i}\times P_{j}\times \;M_{k}\right)+\varepsilon_{ijk} \end{array}$$

where $Y_{ijk}$ is bacterial enumeration in Log CFU/g for the i^t^ salt concentration level, *j*th pH level, and *k*th method, $\;\beta_{0}$ is the overall intercept, $S_{i}$ is the salt concentration, $P_{j}$ is the pH level, $\left(S_{i}\;\times P_{j}\right)$ is the salt by pH interaction; $\beta_{1},\beta_{2},\beta_{12}$ are the corresponding regression coefficients for salt, pH, and the salt by pH interaction, respectively, $\beta_{3}$ M*_k_* is the effect of the *k*th method, and $\beta_{13},\beta_{23},\beta_{123}$ are the regression coefficients for the interactions between salt concentration and method, pH level and method, and between salt concentration, pH level and method, respectively. $W_{ij}$ represents the whole‐plot error with the assumption $W_{ij}∼N\left(0,\sigma_{ij}^{2}\right)$ and $\varepsilon_{ijk}$ is the random residual or split‐plot error term with the assumption $\varepsilon_{ijk}∼N\left(0,\sigma^{2}\right)$, where $W_{ij}$ and $\varepsilon_{ijk}$ are assumed to be independent. The model terms only include the linear effect, and the whole plot error captures the variability contributing from the linear by quadratic and quadratic by quadratic interactions involving salt concentration and pH level, and the quadratic terms for salt concentration and pH level. The residual term captures all other higher‐order interactions. For implementing the model described above and analyzing the data, SAS 9.4 (SAS Institute Inc., 2018) was used. The contour plots were created using R (Version 4.5.3).

## 3 Results and Discussion

Pancreatin powder samples containing, on average, a sodium concentration of 5.5 mg/g and a pH of 6.0 were used in the experiments described here. Based on the results of bacterial enumeration using standard plate methods, the pancreatin powder samples were divided into two groups: with high and low microbial loads. Samples that were part of each group were then combined and homogenized. Subsets from each group underwent analysis for aerobic plate counts before their salt content and pH were adjusted to confirm their levels of microbial contamination using all three methods under evaluation. The results for these enumerations are presented in Table 3.

**TABLE 3:** Initial aerobic plate count results for samples in the high and low microbial contamination groups before salt content and pH were adjusted.

		Method		
Group	Sub‐sample	Petrifilm AC (Log_10_ CFU/g)	Pour plate (Log_10_ CFU/g)	Spread plate (Log_10_ CFU/g)
High microbial Load	1	4.03	4.23	4.08
	2	4.06	3.99	4.01
	3	4.10	4.07	4.13
	Average	4.06	4.10	4.07
Low microbial load	1	2.82	2.83	2.81
	2	2.84	2.82	2.80
	3	2.81	2.93	2.89
	Average	2.82	2.86	2.83

From Table 3, the average APC for the samples with high microbial load using 3M Petrifilm, pour plate, and spread plate methods were 4.06, 4.10, and 4.07 log CFU/g, respectively. These values were found not to be significantly different (*p* > 0.05), and the average microbial load from the three methods for this group was determined to be 4.07 log CFU/g. Similarly, for the low microbial load group, the average enumeration value was 2.83 log CFU/g (*p* > 0.05).

To evaluate the performance of different aerobic bacteria enumeration methods in samples containing high salt concentrations and low pH values, pancreatin powder samples representing products with high and low microbial contamination were used. The salt concentration and pH adjustments were done based on calculations and estimates from preliminary experiments. Once samples were prepared, analyses were then carried out to confirm the salt and acid levels in each of the sub‐samples used for the experimental design, and these results are shown in Table 4.

**TABLE 4:** Target and measured values (average and standard deviation (SD)) for salt and pH on samples prepared for the experiments. Sodium values measured in the samples are also reported here. Measured NaCl concentrations differed slightly from the target concentrations. Statistical analyses were conducted using the measured values.

Target value		Measured values					
NaCl (mg/g)	pH	NaCl (mg/g)		pH		Sodium (mg/g)	
		Average	SD	Average	SD	Average	SD
25.0	4.5	33.37	4.24	4.52	0.06	13.31	0.46
68.0	3.4	62.22	4.74	3.41	0.02	24.89	1.89
68.0	5.6	68.00	2.97	5.53	0.04	27.20	1.19
162.5	3.0	117.19	4.81	2.88	0.04	48.21	4.14
162.5	4.5	143.36	4.72	4.43	0.12	57.49	2.14
162.5	6.0	159.92	4.14	5.96	0.07	59.36	4.68
257.0	3.4	195.72	2.49	3.41	0.06	78.17	0.98
257.0	5.6	219.23	4.69	5.46	0.13	89.03	3.95
300.0	4.5	234.21	3.36	4.54	0.06	95.02	3.36

*N* = 3.

The deviations between the target and measured NaCl concentrations shown in Table 4 were observed during the preparation and verification of the experimental matrices. During preliminary trials, pH and NaCl adjustments were performed based on the assumption that the addition of NaCl and citric acid would have minimal impact on achieving the desired target values. However, it was subsequently observed that adjustments to pH and salt concentration were more complex than anticipated, particularly at higher NaCl levels. The combined effects of the pancreatin matrix, increased ionic strength, and changes in physicochemical properties likely influenced the accuracy of pH and salt adjustments, resulting in deviations from the intended target values. Although efforts were made to achieve the specified pH and NaCl concentrations, some treatments did not reach the exact target values, particularly at higher salt concentrations. Therefore, the actual measured pH and NaCl values were used in all statistical analyses, and contour plots rather than the nominal target values. The measured values more accurately represent the conditions experienced by the microorganisms and provide a more reliable basis for evaluating the effects of pH and salt concentration on microbial recovery.

Measured NaCl concentrations differed slightly from the target concentrations. Statistical analyses were conducted using the measured values.

### 3.1 Effect of Salt and pH on APC Results for Pancreatin Powder Samples With High Microbial Load

The results obtained for APC on samples with high microbial load at various salt concentrations and pH levels using the 3M Petrifilm AC, pour, and spread plate methods are summarized in Table 5.

**TABLE 5:** Aerobic plate count (APC) results (average and standard deviation (SD)) for pancreatin powder samples with high microbial load, as determined by the 3 M Petrifilm AC, pour and spread plate methods.

Target		Measured		APC method					
Salt (mg/g)	pH	Salt (mg/g)	pH	Petrifilm AC (Log_10_ CFU/g)	SD	Pour plate (Log_10_ CFU/g)	SD	Spread plate (Log_10_ CFU/g)	SD
25.0	4.5	33.27	4.52	3.89	0.04	3.94	0.02	3.98	0.03
68.0	3.4	62.22	3.41	3.60	0.11	3.64	0.05	3.67	0.03
68.0	5.6	68.00	5.53	3.94	0.04	3.83	0.02	3.85	0.02
162.5	3.0	117.19	2.88	3.48	0.00	3.34	0.06	3.45	0.03
162.5	4.5	143.36	4.43	3.42	0.03	3.64	0.04	3.74	0.03
162.5	6.0	159.92	5.96	3.73	0.05	3.81	0.03	3.85	0.01
257.0	3.4	195.72	3.41	3.60	0.11	3.59	0.03	3.61	0.03
257.0	5.6	219.23	5.46	3.64	0.06	3.77	0.03	3.75	0.03
300.0	4.5	234.21	4.54	3.30	0.10	3.49	0.05	3.56	0.02

*N* = 3.

The results obtained by each evaluated method were used to create contour plots that illustrate the influence of pH and salt concentration on APC. The plots for 3M Petrifilm AC, pour, and spread plate methods are shown in Figure 1, and they demonstrate that in samples with high microbial load, both salt concentration and pH had significant linear effect contributions that influence the results obtained by all three methods (*p*‐values = 0.0487 and 0.0001, respectively) when enumerating aerobic bacteria in foods. Based on the contour plots from the fitted model, higher APC results were obtained when the pH values were higher and the salt concentration was lower. Additionally, no statistical differences were observed among the methods (*p*‐value = 0.0820).

**FIGURE 1:**
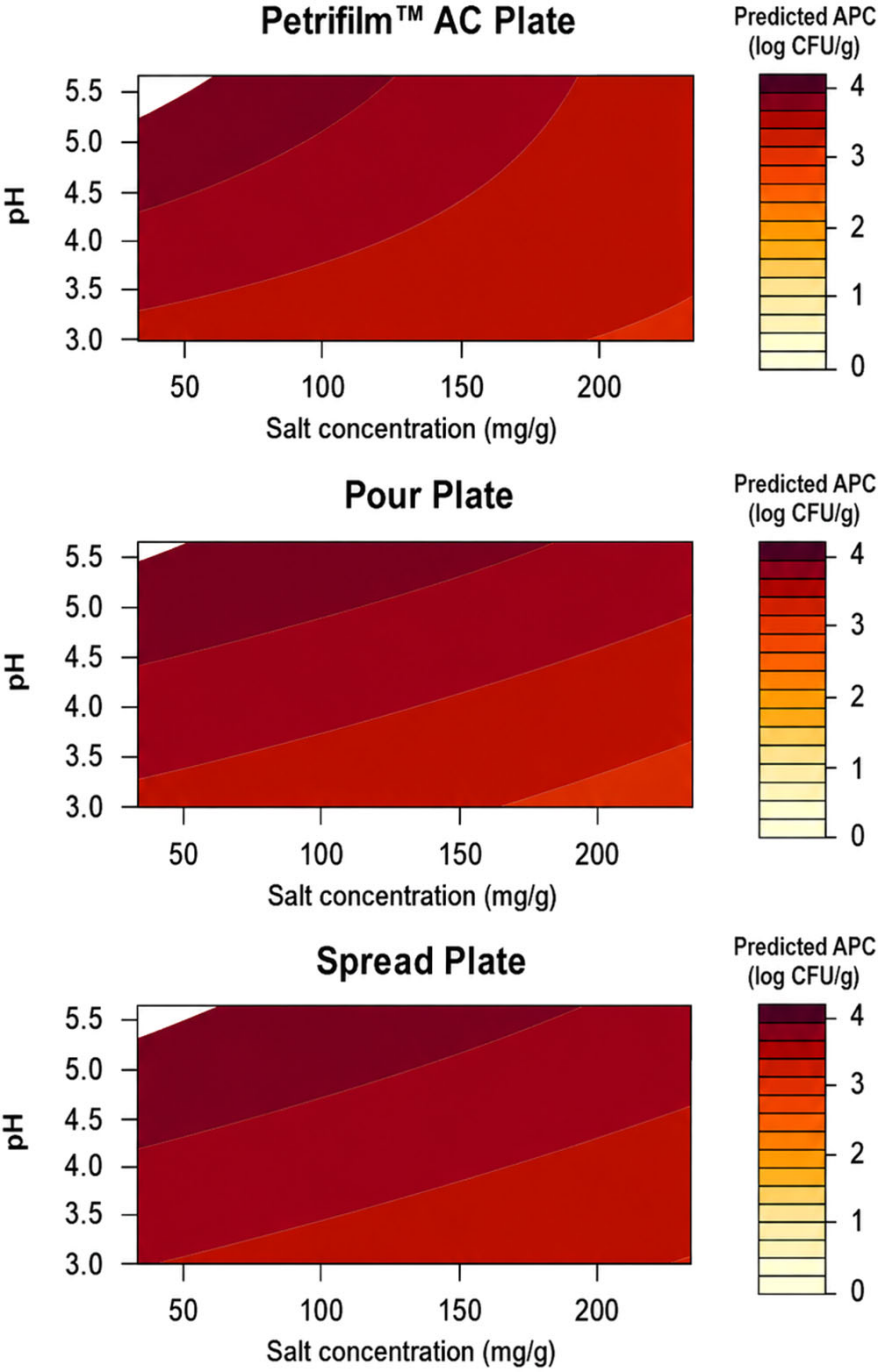
Contour plot showing the influence of salt concentration and pH of pancreatin powder samples on the results for APC when using Petrifilm AC plate, pour plate, and spread plate methods in samples with high microbial load. The contour lines represent predicted log CFU/g values; darker areas indicate higher bacterial counts.

### 3.2 Effect of Salt and pH on APC Results for Pancreatin Powder Samples with Low Microbial Load

The results obtained for APC on samples with low microbial load at different salt concentrations and pH levels, as obtained by the 3M Petrifilm AC, pour, and spread plate methods, are shown in Table 6.

**TABLE 6:** Aerobic plate count (APC) results for pancreatin powder samples with low microbial load, as determined by the 3 M Petrifilm AC, pour and spread plate methods.

Target		Measured		APC method					
Salt (mg/g)	pH	Salt (mg/g)	pH	Petrifilm AC (Log_10_ CFU/g)	SD	Pour plate (Log_10_ CFU/g)	SD	Spread plate (Log_10_ CFU/g)	SD
25.0	4.5	33.27	4.52	2.78	0.16	2.77	0.04	2.72	0.03
68.0	3.4	62.22	3.41	2.70	0.09	2.73	0.05	2.69	0.02
68.0	5.6	68.00	5.53	2.77	0.03	2.76	0.02	2.72	0.05
162.5	3.0	117.19	2.88	0.70	0.00	2.30	0.00	2.23	0.08
162.5	4.5	143.36	4.43	2.65	0.11	2.66	0.07	2.69	0.06
162.5	6.0	159.92	5.96	2.83	0.02	2.70	0.04	2.81	0.07
257.0	3.4	195.72	3.41	0.70	0.00	2.11	0.07	2.18	0.04
257.0	5.6	219.23	5.46	2.69	0.03	2.75	0.04	2.71	0.03
300.0	4.5	234.21	4.54	0.70	0.00	2.00	0.30	2.11	0.10

*N* = 3.

Results obtained from the methods under evaluation were used to create contour plots that show the influence of pH and salt concentration on aerobic bacterial counts in pancreatin powder. The plots for 3M Petrifilm AC, pour, and spread plate methods in samples with low microbial loads are shown in Figure 2. The statistical analysis at low microbial load indicates that the three methods—3M Petrifilm AC, pour plate, and spread plate—show significant differences in terms of APC enumeration at varying quantities of salt concentration and pH. The linear contributions of salt concentration and pH have significant two‐way interactions with method (*p*‐value = 0.0266 and *p*‐value = 0.0016, respectively). The three‐way interaction between salt, pH, and method is not significant (*p*‐value = 0.1323). At the average values of salt and pH, the pour and spread plating methods had higher bacterial enumeration than 3M Petrifilm by 0.4733 Log CFU (95% CI: 0.1671, 0.7796) and 0.4822 Log CFU (95% CI: 0.1760, 0.7884), respectively.

**FIGURE 2:**
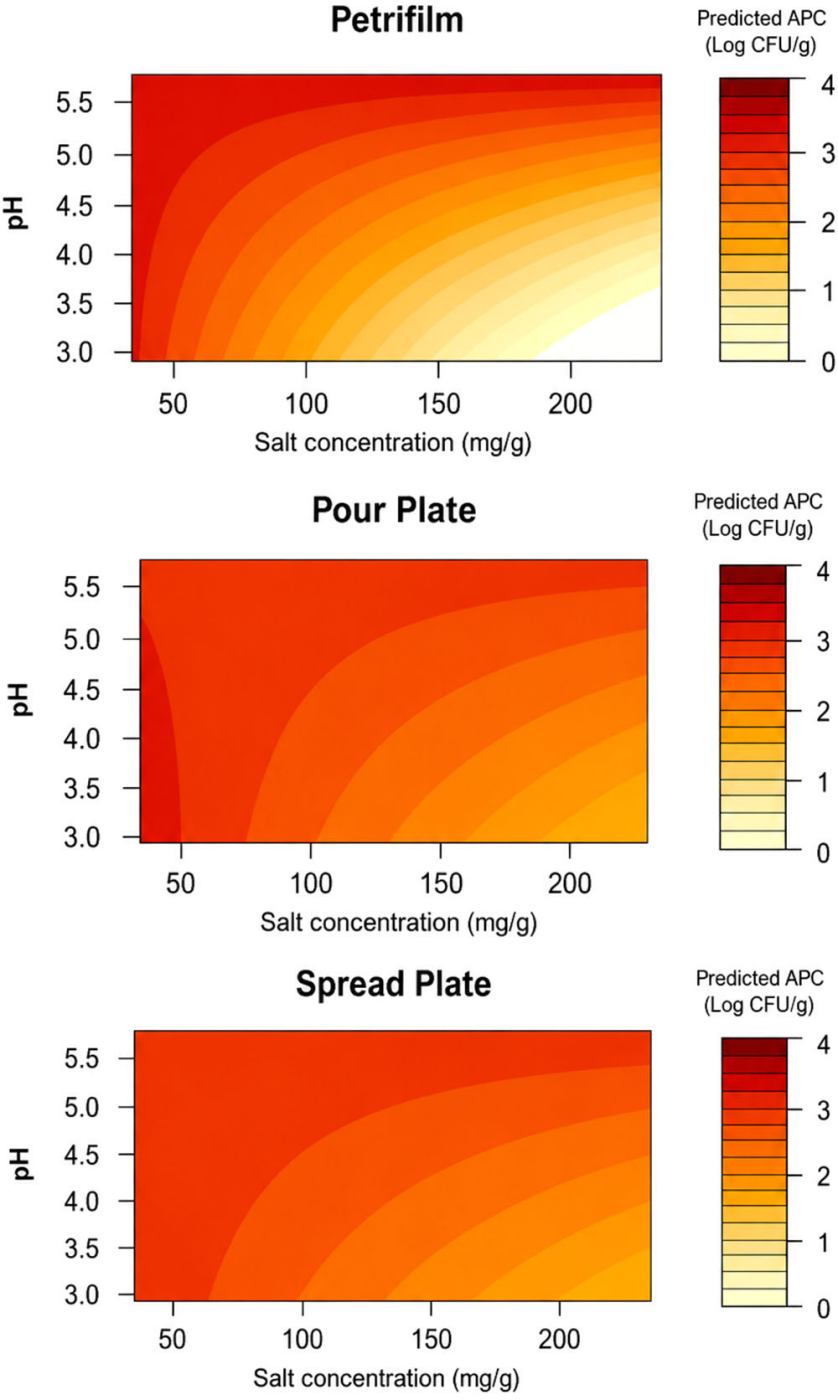
Contour plot showing the influence of salt concentration and pH of pancreatin powder samples on the results for APC when using the Petrifilm AC plate, pour plate and spread plate methods in samples with low microbial load. The contour lines represent predicted log CFU/g values; darker areas indicate higher bacterial counts.

The significant two‐way interaction between pH and method indicates that conclusions cannot be drawn about the effect of salt, pH, and method independently without considering those interactions. For example, when comparing the methods in terms of bacterial enumeration, one must also specify the levels of salt and pH being considered, as the results can vary depending on the levels of these variables. Therefore, the contour plots help determine the combined effects of salt concentrations and pH levels for each method on bacterial enumeration. Figure 2 demonstrates that at low microbial loads, when the salt concentration in the samples was lower, the enumeration values obtained using the different methods were higher regardless of the pH levels. At higher salt concentrations, the effect of the pH was pronounced, and bacterial enumeration increased with an increase in pH value. Once again, based on the contour plots, the pour plate and spread plate methods are more tolerant to changes in pH and salt concentrations than the 3M Petrifilm AC method.

The susceptibility of the 3M Petrifilm AC method to salt concentration and pH is likely related to its lower dilution and buffering capacity compared with the pour and spread plate methods. In the pour plate method, 1 mL of sample is mixed with approximately 15 mL of molten agar, substantially diluting inhibitory compounds and helping neutralize pH. In contrast, the 3M Petrifilm AC method uses only 1 mL of sample to rehydrate the dehydrated medium, so there is little dilution effect. Furthermore, the indicator dye used in 3M Petrifilm AC plates may be affected by pH, potentially reducing colony visibility and resulting in lower perceived counts (3M Technical Bulletin #TB.277375, 2018).

These differences were most apparent in samples with low microbial loads. Because fewer serial dilutions were required, the original salt concentration and pH had greater influence on microbial recovery. In contrast, samples with high microbial loads required additional serial dilutions, which resulted in reduced salt concentrations and moderated pH, minimizing differences among the three enumeration methods.

One other aspect to consider is the passive transport process of salt, known as diffusion, which may occur when high salt concentrations from the plated aliquot move from the surface of the agar plate downwards into the medium, where salt concentration is lower. This process could reduce the salt concentration on the surface of the agar, allowing for bacterial growth when the spread plate method is used, which may help explain why this method outperforms the others evaluated in this study. This diffusion process would not occur with the pour plate or 3M Petrifilm AC methods.

Salt affects the growth of microorganisms by binding water molecules and reducing their availability for microbial growth. When dilution water is added to samples containing salt, it creates a saline environment, which can lead to osmotic shock in microbial cells, ultimately resulting in cell death or growth inhibition (Davidson et al. 2012). However, microorganisms that can survive in high saline conditions have mechanisms that allow them to adapt by adjusting their internal osmolarity. During this adaptation process, the cell takes up compatible solutes, which help maintain the turgor pressure of the cell (Gregory et al., 2021).

pH is a critical parameter that profoundly impacts the growth of organisms. It is determined by the concentration of protons (hydrogen ions, H+) in a solution, with higher proton concentrations indicating lower pH levels (Aoi and Marunaka 2014). pH influences the growth and survival of microorganisms in several ways. Mainly, it determines the environmental conditions suitable for different groups of bacteria. Acidophiles, alkaliphiles, and neutrophiles require varying pH ranges for survival. Acidophiles thrive at pH levels lower than 5, neutrophiles prefer pH between 5 and 9, while alkaliphiles survive best at pH levels higher than 9 (Baker and Dopson 2007). Consequently, microorganisms require specific pH conditions for their growth and survival, and deviations from these conditions may lead to their death or hindered growth.

Even minor pH changes can significantly impact the metabolic activity of microbial communities, with a one‐unit pH change potentially reducing activity by up to 50 percent (Kotsyurbenko et al., 2004). In the context of APC methods, the pH in the growth medium becomes an important factor influencing the microbial community's growth and survival. Alterations in pH can affect the ionization of amino acid functional groups and disrupt hydrogen bonding, leading to changes in molecular folding, denaturation, and ultimately, microbial destruction (Lund et al. 2020). Acidity can further interfere with cellular metabolic processes, causing cell death by affecting the permeability of cell membranes and walls, disrupting cell structure, and interfering with essential cellular functions like ion exchange, metabolism, and protein synthesis (Jin & Zhou, 2022).

Examples of low‐water activity ready‐to‐eat food products that may contain high salt and low pH levels include spice blends. Because these products have very low water activity, the salt and acid present in their formulation may not adversely affect the microorganisms present as they would in a hydrated environment (Beuchat et al. 2013). In harsh environments, such as low water activity, high salt, and low pH, most bacteria, including pathogenic bacteria, overcome various stresses that suppress their ability to grow or survive by forming cysts and spores, altering cellular membranes, expressing repair enzymes for damage, or synthesizing molecules to relieve stress (Haruta and Kanno 2015).

Bacterial membrane proteins are responsible for detecting environmental signals and, when the environment is unsuitable for survival, they promote the production of extracellular cellulose, fimbriae, and changes in membrane permeability (Garmiri et al. 2008). Bacteria also respond to desiccation stress (intense stress due to a loss of water) by expressing enzymes that protect against protein damage and repair DNA damage. This theory suggests that bacteria with robust systems for protecting against protein damage will exhibit better long‐term cell survival. Therefore, this mechanism could also act as a molecular shield against drying stress (Esbelin et al. 2018). Some foodborne pathogens can survive for several months, even years, in low‐water activity foods, making it virtually impossible to eliminate these pathogens in many dry foods or dry food ingredients without impairing their organoleptic quality (Beuchat et al. 2013).

Although APC is not a method designed to detect pathogens, it is effective in quantifying the number of viable microorganisms present in a product. This makes it a valuable tool for assessing sanitation programs and overall quality of the product. Understanding the limitations or disadvantages of each APC method is crucial for selecting an appropriate method for analyzing these challenging matrices.

## 4 Conclusions

Based on the data from this study, the 3M Petrifilm AC method showed lower accuracy in samples containing high salt concentrations and low pH when compared to the pour plate and spread plate methods, likely due to its lower ability to dilute salt and buffer the pH of the final growth medium. Furthermore, based on the statistical analysis of the results obtained in this study, both the spread plate and pour plate methods are acceptable for the enumeration of aerobic bacteria in products containing high salt concentrations and low pH. According to The Compendium of Methods for the Microbiological Examination of Food by the American Public Health Association, these methods are recommended for the enumeration of aerobic bacteria in foods in general (Salfinger and Tortorello 2015).

Understanding the factors that influence the suitability of methods for microbial enumeration in low‐water activity foods with high salt content and low pH levels is crucial for both the food industry and food testing laboratories. The results of this study are essential for supporting the selection of the most effective or appropriate method for assessing the microbiological quality of these food products. By recognizing how these factors impact microbial recovery and enumeration, food processors and analytical laboratories can make informed decisions, ensuring the accuracy and reliability of their testing procedures and, ultimately, contributing to the safety and quality of food products.

Limitations of this study include the use of a single model food (pancreatin powder) and the absence of inter‐laboratory validation. Future research should validate these findings using a broader range of commercial low‐water activity foods, including spice blends, flour, milk powder, and other dry ingredients. Inter‐laboratory studies are also needed to evaluate the reproducibility and robustness of the methods across different testing environments. In addition, future work should investigate the influence of other food matrix characteristics, such as fat, protein, sugar content, and water activity, as well as the recovery of stressed or desiccation‐adapted microorganisms, to further optimize aerobic plate count methods for low‐water activity foods.

Overall, this study demonstrates that the performance of aerobic plate count methods can vary significantly in low‐water activity foods containing high salt concentrations and low pH conditions. Selection of an appropriate enumeration method should consider its dilution capacity and buffering ability to mitigate matrix‐related stresses and ensure accurate microbial recovery.

## Author Contributions

**Waraporn Mahlman**: writing – original draft, conceptualization, methodology, formal analysis, data curation. **Jayne Stratton**: supervision, writing – review and editing, conceptualization. **Reka Howard**: software, writing – review and editing. **Tyler Wiederich**: data curation. **Tai Ha**: methodology. **Andreia Bianchini**: conceptualization, writing – review and editing, supervision.

## Conflicts of Interest

The authors declare no conflicts of interest.

## Supporting information




**jfds71502‐sup‐0001‐TableS1‐S2.docx**: Supplementary Materials
