# Supplementary material for: Effects of Salt and pH on Aerobic Plate Count Methods for Low‐Water Activity Foods
Source: J Food Sci. 2026 Nov 11;91(11):e71502. doi: 10.1111/1750-3841.71502 (PMC13755161; doi:10.1111/1750-3841.71502)
Supplement: Supplementary file 1 [file JFDS-91-0-s001.docx]

**Supplementary Tables:**

**Table S1.** Type of food matrices and environmental samples that have been validated for the use of 3M Petrifilm^TM^ AC Plate

| **Category** | **Type of Foods** |
| --- | --- |
| Meat product | Raw meat (beef, turkey, chicken, pork), ready-to-reheat meat, delicatessen meat |
| Dairy products | Raw milk, powdered milk, powdered products, pasteurized milk, dairy-based desserts, frozen milk |
| Seafood products | Fresh (fish, shrimp), frozen seafood, ready-to-eat, ready-to-reheat seafood |
| Fish and shrimp (Freshwater) | Fresh and frozen (fish, shrimp) |
| Fruits and vegetables | Frozen fruits and vegetables, Fresh and bagged products, ready-to-eat, ready-to-reheat fruits and vegetables |
| Egg products and egg-based products | Pasteurized egg, pastry, mayonnaise, custard |
| Spices | Pepper, thyme, onion powder |
| Flour | White, rye, and wheat flour |
| Pasta | Fresh pasta |
| Nut Halves | Walnut halves |
| Pet food | Dehydrated product (pellets), humid product (balls, pates, sausages), ingredients (dehydrated proteins, dehydrated raw materials) |
| Environmental samples | Surfaces (wipes, swabs), process water, air sampling |

**Table S2**. Validation of 3M Petrifilm^TM^ AC plate against conventional methods.

| **Sample Types** | **Conventional Method** | **Result** | **Reference** |
| --- | --- | --- | --- |
| Water and Wastewater | Membrane filtration method | Significantly comparable | Schraft & Watterworth (2005) |
| Hard-surface carriers  (Stainless steel and porcelain) | Standard plate count on tryptic soy agar (TSA) | Significantly comparable | Nelson et al. (2013) |
| Raw milk | Pour plate method | Significantly comparable | Byrne & Bishop (1991) |
| Milk from cows with clinical mastitis | Pour plate method | Significantly comparable | McCarron et al. (2009) |
| Dehydrated onion and garlic samples | AOAC 966.23 Pour plate method | Significantly difference | Feng et al. (2017) |
| Ready-to-eat foods (sandwiches, cooked meats, cooked pies, milk, dairy products, prepared vegetables, salads, sauces, and cakes) | Spiral plate method | From 113 samples, 86% yielded comparable results, and 14% showed a significant difference | Ellis & Meldrum (2002) |
| Raw and pasteurized milk | Pour plate method at different incubation conditions IDF/ISO, APHA | Significantly comparable | Freitas et al. (2009) |
| Yogurt Starter Cultures  (*Streptococcus salivarius* subsp.  *Thermophilus* and *Lactobacillus delbrueckii* subsp. *Bulgaricus)* | ISO 9232 | Significantly comparable | Gonçalves et al. (2009) |
| Biofilm Bacteria (*Pseudomonas aeruginosa* biofilm) | Drop plated on R2A agar plates | Significantly comparable | Fritz et al. (2015) |
| Colostrum from cow | Spread plate method | Significantly comparable | Morin et al. (2021) |
| Sterilized Sheep milk | Pour plate method | Significantly comparable | De Souza et al. (2015) |
| Meat sample (pork, Chicken, beef) | Pour plate method | Significantly comparable | Park et al. (2001) |
| *Bacillus sporothermodurans* and *Geobacillus stearothermophilus* in ultra-high temperature milk | Pour plate and spread plate method | Significantly comparable | Casillas-Buenrostro et al. (2012) |
| Alfalfa sprouts, beef, chicken, corn meal, pasta flour, pork loin chops, pork rib chops, salmon, scallops, wheat cereal, chocolate ice cream, chocolate milk, nonfat dry milk, soft pasteurized cheese brie, raw milk, whole milk-pasteurized | Pour plate method | Significantly comparable | Garry et al. (2004) |
| Crottin goat’s cheese | Pour plate method | Significantly comparable | De Sousa et al. (2005) |
| Fresh whiting fish, whole and fillets | Pour plate method | Significantly comparable for fillet fish but significantly higher in whole fish | Abgrall & Cleret. (1990) |
